# Clinical impact of lymphocyte/C-reactive protein ratio on postoperative outcomes in patients with rectal cancer who underwent curative resection

**DOI:** 10.1038/s41598-022-21650-1

**Published:** 2022-10-13

**Authors:** Takehito Yamamoto, Meiki Fukuda, Yoshihisa Okuchi, Yoshiki Oshimo, Yuta Nishikawa, Koji Hisano, Takayuki Kawai, Kohta Iguchi, Yukihiro Okuda, Ryo Kamimura, Eiji Tanaka, Hiroaki Terajima

**Affiliations:** Department of Gastroenterological Surgery and Oncology, Kitano Hospital Medical Research Institute, 2-4-20, Ogimachi, Kita-ku, Osaka, 530-8480 Japan

**Keywords:** Gastroenterology, Oncology

## Abstract

Cancer-related systemic inflammation influences postoperative outcomes in cancer patients. Although the relationship between inflammation-related markers and postoperative outcomes have been investigated in many studies, their clinical significance remains to be elucidated in rectal cancer patients. We focused on the lymphocyte count/C-reactive protein ratio (LCR) and its usefulness in predicting short- and long-term outcomes after rectal cancer surgery. Patients with rectal cancer who underwent curative resection at our institution between 2010 and 2018 were enrolled in this study. We comprehensively compared the effectiveness of 11 inflammation-related markers, including LCR and other clinicopathological characteristics, in predicting postoperative complications and survival. Receiver operating characteristic curve analysis indicated that LCR had the highest area under the curve value for predicting the occurrence of postoperative complications. In the multivariate analysis, male sex (odds ratio [OR]: 2.21, 95% confidence interval [CI] 1.07–4.57, *P* = 0.031), low tumor location (OR: 2.44, 95% CI 1.23–4.88, *P* = 0.011), and low LCR (OR: 3.51, 95% CI 1.63–7.58, *P* = 0.001) were significantly and independently associated with the occurrence of postoperative complications. In addition, multivariate analysis using Cox’s proportional hazard regression model for the prediction of survival showed that low LCR (≤ 12,600) was significantly associated with both poor overall survival (hazard ratio [HR]: 2.07, 95% CI 1.03–4.15, *P* = 0.041) and recurrence-free survival (HR: 2.21, 95% CI 1.22–4.01, *P* = 0.009). LCR is a useful marker for predicting both short- and long-term postoperative outcomes in rectal cancer patients who underwent curative surgery.

## Introduction

Colorectal cancer is one of the most common cancers worldwide. Surgery is the standard treatment for resectable colorectal cancer. Although the postoperative outcome of colorectal cancer has improved, preventing postoperative complications remains challenging. Anastomotic leakage and urogenital and sexual dysfunctions are common complications after rectal surgery, and the risk is much higher than that associated with colon surgery. Postoperative complications can result in increased overall inpatient costs and sometimes lead to delayed initiation of postoperative treatments, including adjuvant chemotherapy. Furthermore, they negatively affect long-term survival^1^. Therefore, determining a biomarker to identify patients at a high risk of postoperative complications is important.

Cancer-related systemic inflammation was first reported by Rudolf Virchow in 1863^2^. Inflammatory responses can proliferate residual cancer cells and stimulate micrometastases, leading to a risk of recurrence. Recently, preoperative systemic inflammation has been reported to be associated with poor prognosis after surgery in several types of cancer, including colorectal cancer. A review of previous research showed that combinations of inflammation-related variables, including serum neutrophil count, lymphocyte count, monocyte count, platelet count, C-reactive protein (CRP) concentration, and albumin concentration, the values of which are available from preoperative routine blood examinations can be useful biomarkers^3^.

CRP is a useful preoperative and postoperative parameter that reflects the inflammatory status in patients and is routinely evaluated in patients undergoing surgery in Japan. The combinations of CRP and other inflammatory variables, namely CRP/albumin ratio (CAR) and lymphocyte count/CRP ratio (LCR), have been reported to affect short-term and long-term postoperative outcomes^4–13^. However, only few studies have focused on patients with rectal cancer who underwent curative resection; moreover, the optimal cut-off value of each biomarker for rectal cancer has not been thoroughly scrutinized.

In the present study, we comprehensively compared the potential of various inflammation-related markers to predict postoperative short- and long-term outcomes in patients with rectal cancer who underwent curative resection. Furthermore, we evaluated the relationship between LCR and postoperative outcomes.

## Materials and methods

### Patients

A total of 202 patients with rectal cancer who underwent curative surgery at Kitano Hospital between 2010 and 2018 were enrolled in this study. Patients who underwent non-curative resection, recurrent tumor resection, or had distant metastasis were excluded. The tumor was located below the lower margin of the second sacral vertebra in all the patients. Based on tumor location, patients were categorized into the high (above the peritoneal reflection) and low (below the peritoneal reflection including the anal canal) groups. The clinical stage was determined using colonoscopy, computed tomography (CT), magnetic resonance imaging, and contrast-enhanced colonography.

### Treatment protocol

Treatment strategies, including surgical procedures and perioperative chemotherapy or chemoradiotherapy, were determined for individual cases by a multidisciplinary team. Neoadjuvant chemoradiotherapy or neoadjuvant chemotherapy was administered in cases where there was high risk for recurrence, such as for bulky tumors and swelling of multiple lymph nodes. All surgeries were performed or managed by board-certified colorectal surgeons.

### Postoperative follow-up

Patients were postoperatively followed up according to the guidelines of the Japanese Society for Cancer of the Colon and Rectum^14^; physical examination and blood tests were performed every 3 months in the first 3 years and every 6 months thereafter. CT was performed every 6 months during the first 3 years after the operation and every year thereafter. Overall survival (OS) was defined as the period from the date of surgery to the date of death from any cause, and recurrence-free survival (RFS) as that from the date of surgery to the date of recurrence or death from any cause.

### Inflammation-related markers

We reviewed the following 11 inflammation-related markers: lymphocyte count (/µL)/CRP (mg/dL) ratio (LCR), lymphocyte count/monocyte count (/µL) ratio (LMR), monocyte count (/µL)/albumin (g/dL) ratio (MAR), neutrophil count (/µL)/albumin (g/dL) ratio (NAR), neutrophil count/lymphocyte count (/µL) ratio (NLR), platelet count (/µL)/albumin (g/dL) ratio (PAR), platelet count/lymphocyte count (/µL) ratio (PLR), CRP (mg/dL)/albumin (g/dL) ratio (CAR), prognostic nutritional index (PNI), Glasgow prognostic score (GPS), and systemic inflammation score (SIS). All marker data were generated by routine preoperative blood examinations conducted within 2 weeks before the operation.

Regarding GPS, patients with both high serum CRP concentration (> 1.0 mg/dL) and low albumin concentration (< 3.5 g/dL) were assigned a score of 2; those with either of these two abnormal values, a score of 1; and those with neither, a score of 0^15^. Regarding SIS, patients with both low albumin concentration (< 4.0 g/dL) and low LMR (< 4.44) were assigned a score of 2; those with either of these two abnormal values, a score of 1; and those with neither, a score of 0^16,17^. PNI was calculated using the following formula: albumin concentration (g/L) + 0.005 × lymphocyte count (/µL)^18^

### Prediction of postoperative complications

Postoperative complications were categorized according to the Clavien-Dindo classification^19^. We analyzed the correlation between the values of preoperative inflammation-related markers and the occurrence of postoperative complications (≥ Clavien-Dindo grade II) and compared the predictive performance of the markers with other clinical characteristics of the patients. The optimal cut-off value of each inflammation-related marker was determined using receiver operating characteristic (ROC) curve analysis.

### Prognostic analysis

For the prognostic analysis, we focused on LCR. The patients were divided into high LCR and low LCR groups. We compared the clinical impact of LCR and other clinicopathological characteristics (such as age, sex, tumor location, carcinoembryonic antigen [CEA], pT category and pN category, and histology) on OS and RFS.

### Statistical analysis

Continuous variables were presented as median [range] or mean ± standard deviation. The Fisher’s exact test or chi-square test was used to compare categorical variables. Prognostic analysis was performed using the log-rank test and the Kaplan–Meier method. Variables with a *P* value < 0.05 in the preceding univariate analysis were subjected to multivariate analysis using the Cox’s proportional hazard regression model. All analyses were two-sided, and a *P* value < 0.05 was considered statistically significant. All statistical analyses were performed using JMP Pro, version 16 (SAS Institute Inc., Cary, NC, USA).

### Ethics approval

This retrospective study protocol was approved by the institutional review board of Kitano Hospital (reference no. 2201003) and conformed to the provisions of the Declaration of Helsinki.

### Consent to participate

Informed consent was obtained in the form of opt-out on the website. Those who rejected were excluded.

## Results

### Patient characteristics and outcomes

**Figure 1:**
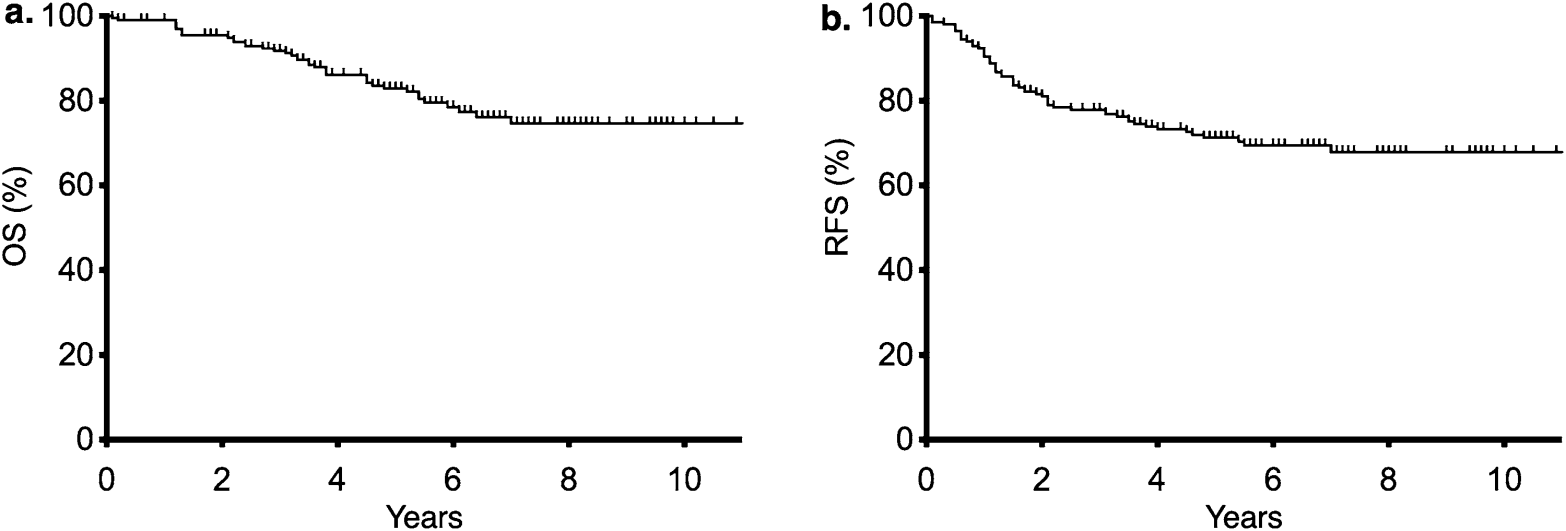
(**a**) Overall survival (OS) and (**b**) recurrence-free survival (RFS) of all study participants.

The clinicopathological characteristics of the study participants are shown in Table 1. A total of 202 patients (121 men and 81 women) with a median age of 67 years (range 34–93 years) were included. The mean body mass index was 22.4 ± 3.5 kg/m^2^. The tumor location was high in 98 patients (49%) and low in 104 patients (51%). Postoperative complications ≥ Clavien-Dindo grade II occurred in 52 patients (infectious complications that required the use of antibiotics in 26 patients, anastomotic leakage in 10, ileus in 9, anastomotic bleeding in 4, and other complications in 6). Pathologically positive regional lymph node metastases were observed in 56 patients (28%). Table 2 lists the test results of inflammation-related variables. The median follow-up duration was 62.8 months. The 5-year OS and RFS rates of all 202 patients were 82.9% and 70.6%, respectively (Fig. 1).

**Table 1:** Clinicopathological characteristics of study participants.

Variables	Median [range] or N (%)
Age (years)	67 [34–93]
**Sex**	
Male	121 (60)
Female	81 (40)
BMI (kg/m^2^)	22.4 ± 3.5
**Tumor location**	
High	98 (49)
Low	104 (51)
**Operative approach**	
Open	22 (11)
Laparoscopic	180 (89)
**cT classification**	
cT1–2	85 (42)
cT3–4	117 (58)
**cStage**	
I	67 (33)
II	51 (25)
III	84 (42)
CEA (ng/mL)	3.4 [0.7–332.5]
**Preoperative treatment**	
None	167 (83)
nCRT	29 (14)
NAC	6 (3)
Operation time (minutes)	301 [53–996]
Blood loss (mL)	25 [0–900]
**Histology**	
Well/mod, papillary	188 (93)
Poor, mucinous	9 (5)
**pT classification**	
pT0–2	101 (50)
pT3–4	101 (50)
**pN classification**	
Positive	56 (28)
Negative	146 (72)
**Complications ≥ Clavien‒Dindo grade II**	
Infection	23 (13)
Anastomotic leakage	10 (6)
Ileus	9 (5)
Anastomotic bleeding	4 (2)
Others	6 (3)
Adjuvant treatment	72 (36)

*BMI* body mass index, *cT* clinical stage based on tumor size and spread of cancer to nearby tissue, *cStage* clinical stage of cancer, *CEA* carcinoembryonic antigen, *nCRT* neoadjuvant chemoradiotherapy, *NAC* neoadjuvant chemotherapy, *well/mod* well-differentiated/moderately-differentiated, *poor* poorly-differentiated, *pT* pathological stage based on tumor size and spread of cancer to nearby tissue, *pN* pathological stage based on spread of cancer to nearby lymph nodes.

**Table 2:** Values of inflammation-related variables of study participants.

Variables	Median [range] or N (%)
Neutrophil count (/µL)	3207 [748–12768]
Lymphocyte count (/µL)	1354 [184–4585]
Monocyte count (/µL)	397 [27–1018]
Platelet count (× 10^3^/µL)	215.5 [62.0–482.0]
Albumin (g/dL)	4.1 [2.6–5.2]
CRP (mg/dL)	0.11 [0.02–2.43]
**Combinations**	
LCR	46,906 [1567–620444]
LMR	3.38 [0.51–18.5]
MAR	98.8 [6.05–275.2]
NAR	758.1 [202.2–2901.8]
NLR	2.34 [0.45–25.95]
PAR	51197 [16429–121538]
PLR	157.4 [32.0–1048.9]
CAR	0.027 [0.004–0.726]
PNI	47.4 [33.3–63.5]
**GPS**	
0	184 (91)
1	14 (7)
2	4 (2)
**SIS**	
0	46 (23)
1	104 (51)
2	52 (26)

*CRP* C-reactive protein, *LCR* lymphocyte count/C-reactive protein ratio, *LMR* lymphocyte count/monocyte count ratio, *MAR* monocyte count/albumin ratio, *NAR* neutrophil count /albumin ratio, *NLR* neutrophil count/lymphocyte count ratio, *PAR* platelet count /albumin ratio, *PLR* platelet count/lymphocyte count, *CAR* C-reactive protein/albumin ratio, *PNI* prognostic nutritional index, *GPS* Glasgow prognostic score, *SIS* systemic inflammation score.

### Comparison among the inflammation-related markers

The study participants were divided into the complication and non-complication groups based on the occurrence of postoperative complications ≥ Clavien-Dindo grade II. We performed ROC curve analysis for continuous variables to determine the optimal cut-off value to predict the occurrence of postoperative complications. The area under the curve value of LCR, LMR, and PNI was 0.57, which was higher than that of any other marker (Supplementary Fig. S1). We compared the values of all the 11 inflammation-related markers between the complication and non-complication groups (Table 3). LCR, LMR, NLR, PAR, CAR, PNI, and GPS were significantly associated with the occurrence of postoperative complications, and further analysis was conducted with a focus on the LCR ≤ 12,600 (*P* < 0.001).

**Table 3:** Univariate analysis showing the association between 11 combinations of inflammation-related markers and occurrence of postoperative complications.

Variables	Complication	No complication	*P* value
**LCR**			
≤ 12,600	18	20	
> 12,600	34	130	< 0.001*
**LMR**			
≤ 3.038	29	55	
> 3.038	23	95	0.016*
**MAR**			
≤ 160	44	140	
> 160	8	10	0.057
**NAR**			
≤ 1086.5	40	129	
> 1086.5	12	21	0.127
**NLR**			
≤ 3.24	31	118	
> 3.24	21	32	0.007*
**PAR**			
≤ 40,750	15	24	
> 40,750	37	126	0.043*
**PLR**			
≤ 208	31	110	
> 208	21	40	0.063
**CAR**			
≤ 0.093	38	131	
> 0.093	14	19	0.017*
**PNI**			
≤ 47.8	35	73	
> 47.8	17	77	0.020*
**GPS**			
0	44	140	
1	4	10	
2	4	0	0.003*
**SIS**			
0	11	35	
1	26	78	
2	15	37	0.830

*LCR* lymphocyte count/C-reactive protein ratio, *LMR* lymphocyte count/monocyte count ratio, *MAR* monocyte count/albumin ratio, *NAR* neutrophil count/albumin ratio, *NLR* neutrophil count/lymphocyte count ratio, *PAR* platelet count/albumin ratio, *PLR* platelet count/lymphocyte count, *CAR* C-reactive protein/albumin ratio, *PNI* prognostic nutritional index, *GPS* Glasgow prognostic score, *SIS* systemic inflammation score. *P < 0.05

### Risk for postoperative complications

Table 4 shows the comparison between LCR and other clinicopathological characteristics in terms of their association with the occurrence of postoperative complications. The univariate analysis showed that male sex, low tumor location, and LCR ≤ 12,600 were significantly associated with the occurrence of postoperative complications. Similarly, in the multivariate analysis, male sex (odds ratio [OR]: 2.21, 95% confidence interval [CI] 1.07–4.57, *P* = 0.031), low tumor location (OR: 2.44, 95% CI 1.23–4.88, *P* = 0.011), and LCR ≤ 12,600 (OR: 3.51, 95% CI 1.63–7.58, *P* = 0.001) were independent risk factors for postoperative complications.

**Table 4:** Univariate and multivariate analyses showing the association of clinicopathological characteristics and LCR with the occurrence of postoperative complications.

Variables	Univariate			Multivariate		
	Complication	No complication	*P* value	OR	95%CI	*P* value
**Age (years)**						
≤ 69	28	95				
> 70	24	55				
**Sex**						
Male	38	83		2.21	1.07–4.57	0.031*
Female	14	67	0.033*			
**BMI (kg/m**^**2**^**)**						
≤ 25	7	36				
> 25	45	114	0.110			
**Location**						
High	17	81				
Low	35	69	0.008*	2.44	1.23–4.88	0.011*
**Operative approach**						
Open	4	18				
Laparoscopy	48	132	0.452			
**CEA (ng/mL)**						
≤ 5	33	99				
> 5	19	51	0.740			
**Preoperative treatment**						
None	38	129				
nCRT	12	17				
NAC	2	4	0.097			
**Operation time (minutes)**						
≤ 300	22	79				
> 300	30	71	0.198			
**Blood loss (mL)**						
≤ 100	33	114				
> 100	16	34	0.177			
**pT classification**						
pT0–2	49	145				
pT3–4	3	5	0.872			
**pN classification**						
Positive	16	40				
Negative	36	110	0.569			
**LCR**						
> 12,600	34	130				
≤ 12,600	18	20	< 0.001*	3.51	1.63–7.58	0.001*

*CI* confidence interval, *BMI* body mass index, *CEA* carcinoembryonic antigen, *nCRT* neoadjuvant chemoradiotherapy, *NAC* neoadjuvant chemotherapy, *pT* pathological stage based on the tumor size and spread of cancer to nearby tissue, *pN* pathological stage based on spread of cancer to nearby lymph nodes, *LCR* lymphocyte count/C-reactive protein ratio. *P < 0.05

### Predictive value of LCR for prognosis

The prognostic effects of the patient characteristics including LCR were investigated (Table 5). During the study period, 39 deaths (19%) and 41 recurrences (20%) occurred.

**Table 5:** Univariate and multivariate analyses showing impact of clinicopathological characteristics and LCR on OS and RFS.

Variables	OS						RFS				
	Univariate			Multivariate			Univariate		Multivariate		
	n	5-year OS (%)	*P* value	HR	95%CI	*P* value	5-year RFS (%)	*P* value	HR	95%CI	*P* value
**Age (years)**											
≤ 69	123	92.7					76.6				
> 70	79	67.6	< 0.001*	2.94	1.49–5.77	0.002*	61.1	0.008*	2.04	1.20–3.47	0.008*
**Sex**											
Male	121	84.6					66.9				
Female	81	80.5	0.909				76.8	0.153			
**Location**											
High	98	86.6					73.1				
Low	104	79.6	0.156				68.2	0.586			
**BMI (kg/m**^**2**^**)**											
≤ 25	159	82.5					70.2				
> 25	43	84.5	0.53				72.0	0.549			
**Operative approach**											
Open	22	65.5					71.1				
Laparoscopy	180	85.2	0.083				66.9	0.495			
**CEA (ng/mL)**											
≤ 5	132	90.8					79.0				
> 5	70	67.8	< 0.001*	1.80	0.88–3.69	0.106	54.8	< 0.001*	1.33	0.77–2.30	0.309
**pT classification**											
pT0–2	101	90.3					86.0				
pT3–4	101	76.1	0.003*	2.22	0.98–5.01	0.056	55.9	< 0.001*	3.00	1.54–5.85	0.001*
**pN classification**											
Negative	146	87.8					79.0				
Positive	56	70.7	0.007*	1.66	0.84–3.28	0.132	49.6	< 0.001*	2.32	1.33–4.03	0.003*
**Preoperative treatment**											
None	167	84.5					72.4				
nCRT	29	78.6					66.7				
NAC	6	66.7	0.111				61.4	0.525			
**Operation time (minutes)**											
≤ 300	101	83.7					74.6				
> 300	101	82.2	0.892				66.7	0.349			
**Blood loss (mL)**											
≤ 100	147	89.5					76.2				
> 100	50	61.1	< 0.001*	2.74	1.38–5.43	0.004*	52.0	0.004*	2.38	1.36–4.18	0.003*
**Histology**											
Well/mod, papillary	188	84.0					71.3				
Poor, mucinous	9	63.5	0.226				63.5	0.713			
**LCR**											
> 12,600	164	84.7					73.0				
≤ 12,600	38	75.2	0.039*	2.07	1.03–4.15	0.041*	60.0	0.025	2.21	1.22–4.01	0.009*

*CI* confidence interval, *HR* hazard ratio, *OS* overall survival, *RFS* recurrence-free survival, *BMI* body mass index, *CEA* carcinoembryonic antigen, *pT* pathological stage based on the tumor size and spread of cancer to nearby tissue, *pN* pathological stage based on spread of cancer to nearby lymph nodes, *well/mod* well-differentiated/moderately-differentiated, *poor* poorly-differentiated, *LCR* lymphocyte count/C-reactive protein ratio. *P < 0.05

**Figure 2:**
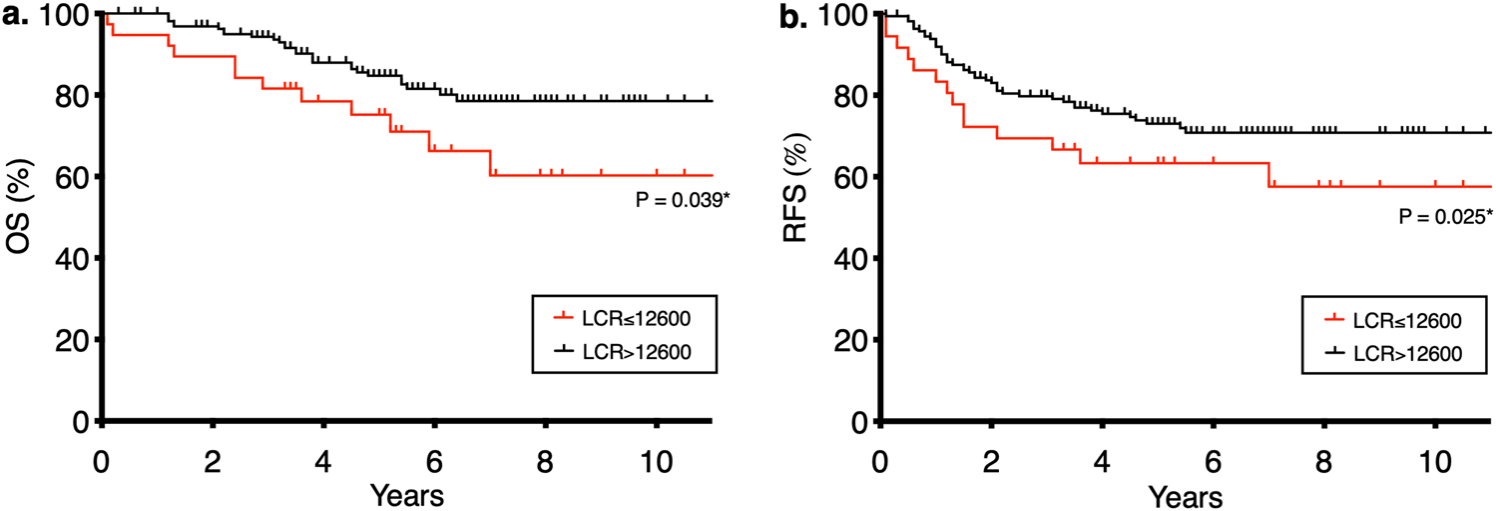
Comparison of (**a**) Overall survival (OS) and (**b**) recurrence-free survival (RFS) between high LCR and low LCR groups. *LCR* lymphocyte count/C-reactive protein ratio.

We first investigated potential prognostic factors for OS (Table 5, left). Univariate analysis showed that age > 70 years, CEA > 5 ng/mL, pT3-4, pN+, intraoperative blood loss > 100 mL, and LCR ≤ 12,600 were significantly associated with poor OS. In multivariate analysis using Cox’s proportional hazard regression model, age > 70 years (hazard ratio [HR]: 2.94, 95% CI 1.49–5.77, *P* = 0.002), intraoperative blood loss > 100 mL (HR: 2.74, 95% CI 1.38–5.43, *P* = 0.004), and LCR ≤ 12,600 (HR: 2.07, 95% CI 1.03–4.15, *P* = 0.041) were significantly associated with poor OS. The Kaplan–Meier curve depicting the effect of LCR on OS is shown in Fig. 2a.

We also investigated potential prognostic factors for RFS (Table 5, right). Univariate analysis revealed that age > 70 years, CEA > 5 ng/mL, pT3-a, pN+, intraoperative blood loss > 100 mL, and LCR ≤ 12,600 were significantly associated with poor RFS. In multivariate analysis, age > 70 years (HR: 2.04, 95% CI 1.20–3.47, *P* = 0.008), pT3–4 (HR: 3.00, 95% CI 1.54–5.85, *P* = 0.001), pN+ (HR: 2.32, 95% CI 1.33–4.03, *P* = 0.003), intraoperative blood loss > 100 mL (HR: 2.38, 95% CI 1.36–4.18, *P* = 0.003), and LCR ≤ 12,600 (HR: 2.21, 95% CI 1.22–4.01, *P* = 0.009) were significantly associated with poor RFS. The Kaplan–Meier curve representing the effect of LCR on RFS is shown in Fig. 2b.

## Discussion

Recently, many reports have indicated the prognostic potential of inflammation-related biomarkers in patients with colorectal cancer. NLR is one of the most representative biomarkers, and the relationship between NLR and postoperative prognosis in patients with resectable colorectal cancer has been analyzed in many studies^20–26^. Similarly, several papers on the analysis of GPS or modified GPS^27–29^, LMR^30–32^, and PLR^32,33^ in the same population have been reported.

In the present study, we revealed that a low LCR (≤ 12,600) could be associated with both short- and long-term outcomes in patients with rectal cancer who underwent curative resection. The number of patients categorized as having low LCR was 38 (19%), and this cut-off value was appropriate for identifying patients with poor outcomes.

A few studies have revealed a relationship between CRP-related parameters and survival in patients with colorectal cancer. For example, Koike et al. reported that preoperative CRP > 0.5 mg/dL was significantly associated with poor prognosis in patients with stage I and II colorectal cancer^34^. There are also several reports on combinations of CRP and other variables. Dolan et al. indicated that CAR > 0.22 was a marker for poor prognosis in their retrospective analysis of the data of 801 patients with colon cancer^6^. Similarly, Ishizuka et al. analyzed the data of 627 patients with colorectal cancer patients and reported that CAR > 0.038 was associated with poor prognosis^4^. In both these studies, the cut-off values were determined using ROC curve analysis.

The prognostic potential of LCR has also been reported. Suzuki et al. analyzed 16 inflammation-related markers, including NLR, LMR, PLR, CAR, PNI, LCR, NAR, MAR, and PAR, in 1303 patients with colorectal cancer^9^. They concluded that an LCR ≤ 12,980 was significantly associated with a poor prognosis after surgery. Similarly, Okugawa et al. also investigated seven combinations and reported that LCR ≤ 6676 was the most significant predictor of survival in patients with colorectal cancer^12^. In both these studies, the cut-off value for LCR was determined using ROC curve analysis. Nakamura et al. categorized 756 patients with unresectable metastatic colorectal cancer into low, intermediate, and high LCR groups^13^. This stratification was significantly correlated with prognosis and a lower LCR independently affects survival.

Lymphocytes can promote cytotoxic immune responses in cases of malignancies. Decreased serum lymphocyte count can reflect impaired immunity to cancer. Some previous studies have indicated that decreased lymphocyte count itself could be associated with poor survival outcomes in patients with colorectal cancer^35–38^. Low LCR indicates a low lymphocyte count and high CRP concentration, which reflects elevated systemic inflammation and decreased immune function.

No reports have focused on the impact of LCR on postoperative outcomes in patients with rectal cancer. The tumor microenvironment and mechanism of tumor progression and metastasis can differ between the colon and rectum; therefore, it is mandatory to focus specifically on rectal cancer to identify the optimal inflammation-related marker and cut-off value. The present study showed that an LCR ≤ 12,600 could be an important biomarker in patients with resectable rectal cancer.

Importantly, no study has analyzed the predictive potential of inflammation-related markers for short-term outcomes after rectal cancer surgery. The rates of postoperative complications, including anastomotic leakage, intra-abdominal infectious disease, and urogenital, sexual, and anal dysfunctions, are generally higher in patients with rectal cancer than those in patients with colon cancer. This can be attributed to the immunological vulnerability of these patients and correlated with preoperative cancer-related systemic inflammation.

Recently, Artinyan et al. indicated a significant relationship between postoperative complications and poor prognosis in a large-scale database study that included 12,075 patients with colorectal cancer^1^. Therefore, it is important to identify biomarkers associated with short-term outcomes in order to improve long-term outcomes. Okugawa et al. reported that a preoperative LCR ≤ 6000 was significantly correlated with the rate of postoperative infectious complications as well as long-term survival, although they enrolled both patients with colon and rectal cancer^7^.

In the present study focusing on patients with rectal cancer, preoperative LCR ≤ 12,600 was significantly and independently associated with the rate of postoperative complications. Therefore, meticulous postoperative management is necessary for patients with a low preoperative LCR in order to improve short-term outcomes.

Preoperative treatments are important for advanced rectal cancer with high risk for recurrence, and 29 patients underwent neoadjuvant chemoradiotherapy (nCRT) and six patients underwent neoadjuvant chemotherapy (NAC) in the present study. In our dataset, there was no significant difference in LCR between patients with and without preoperative treatments (48,888 [1567–620,444] vs. 65,664 [4168–452,676], *P* = 0.162). We consider that LCR can independently affect postoperative outcomes regardless of the preoperative treatments.

To the best of our knowledge, this is the first report to indicate the predictive value of LCR for both short- and long-term outcomes, specifically in patients with rectal cancer. The assessment of preoperative LCR can help physicians identify subpopulations at high risk for postoperative complications and poor prognosis, and this can lead to improvement of short- and long-term outcomes after rectal cancer surgery.

This study has some limitations. First, the data were collected over a long time, and the results might have been affected by chronological changes in pre- and postoperative management, including chemotherapy regimens. Second, the optimal timing of blood sample collection was not evaluated. In the present study, the timing differed within the 2-week period before the operation depending on the patients. It is necessary to standardize the timing of blood tests to establish robust evidence regarding LCR. Third, owing to the retrospective nature of this single-center study involving a relatively small population, there might have been selection bias. Larger studies are necessary to establish the clinical utility of LCR as a biomarker for predicting short- and long-term survival of patients with rectal cancer.

## Supplementary Information

Supplementary Figure S1.

Supplementary Legends.

## Data Availability

The data that support the findings of this study are available on reasonable request from the corresponding author.
