# Supplementary figures and images for: Clinical impact of lymphocyte/C-reactive protein ratio on postoperative outcomes in patients with rectal cancer who underwent curative resection
Source: Sci Rep. 2022 Oct 13;12:17136. doi: 10.1038/s41598-022-21650-1 (PMC9561722; doi:10.1038/s41598-022-21650-1)

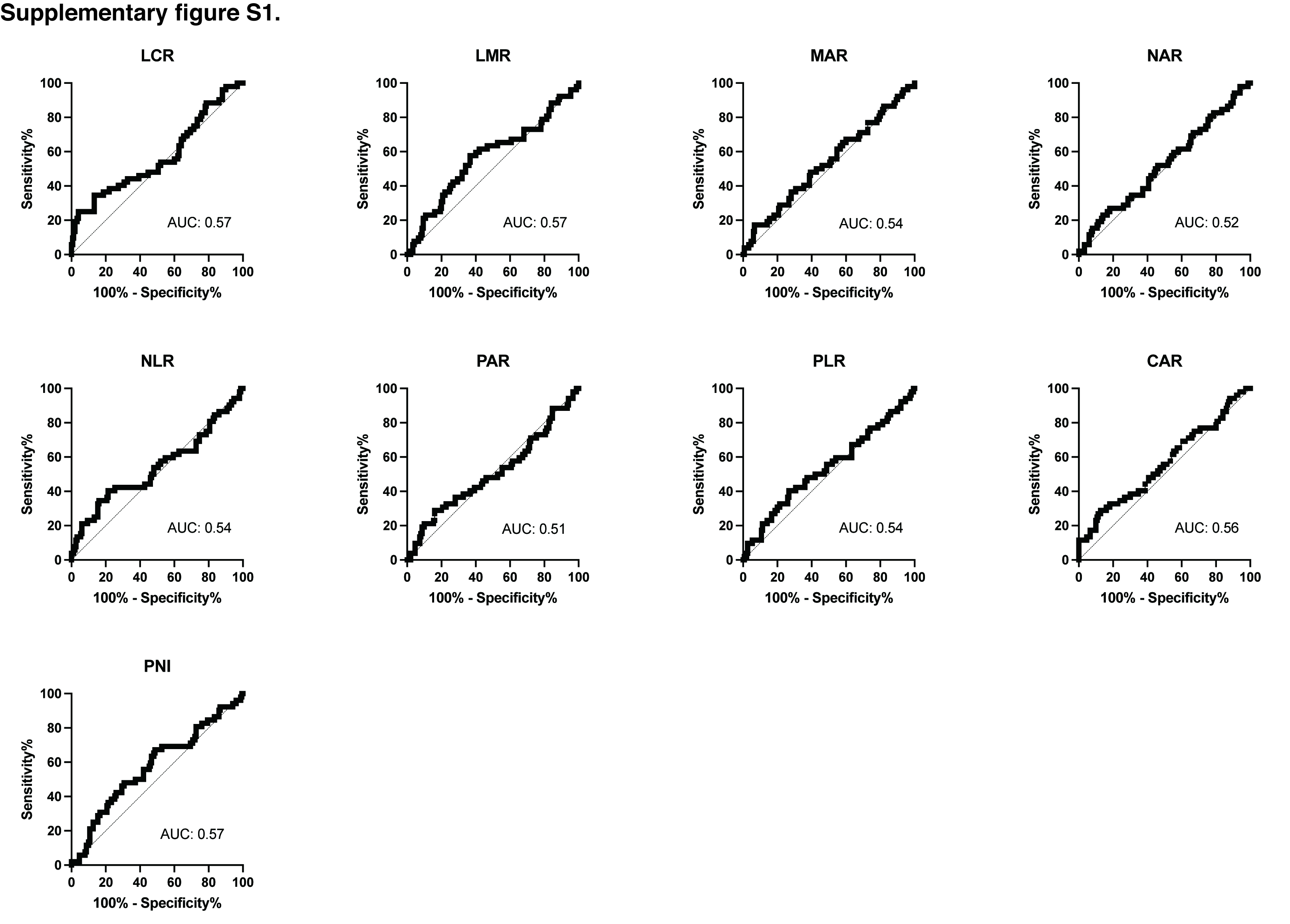

Supplement: Supplementary file 1 — Supplementary Figure S1. [file 41598_2022_21650_MOESM1_ESM.tif]
