# Supplementary material for: Clinical impact of lymphocyte/C-reactive protein ratio on postoperative outcomes in patients with rectal cancer who underwent curative resection
Source: Sci Rep. 2022 Oct 13;12:17136. doi: 10.1038/s41598-022-21650-1 (PMC9561722; doi:10.1038/s41598-022-21650-1)
Supplement: Supplementary file 2 — Supplementary Legends. [file 41598_2022_21650_MOESM2_ESM.docx]

**Supplementary figure legends**

**Supplementary Fig. S1.** Receiver operating characteristic curve analysis to evaluate the predictive value of nine combinations of inflammation-related markers for occurrence of postoperative complications

AUC, area under the curve
